# Multidimensional Quality of Life Among Individuals with Type 2 Diabetes Mellitus: A Cross-Sectional Study, Al Baha, Saudi Arabia

**DOI:** 10.3390/ijerph22121784

**Published:** 2025-11-25

**Authors:** Mohammad A. Albanghali, Shaimaa A. Abdalgeleel, Uthman Albakri, Soltan J. Algamdi, Yasser M. Kofiah, Abdulrahman A. Alghamdi, Saleh Alghamdi, Amer A. Alshehri, Norah A. Alghamdi, Laila A. Alghamdi, Shatha A. Alzahrani, Mashael M. Alzahrani, Batol M. Albanghali, Basim A. Othman

**Affiliations:** 1 Department of Public Health, Faculty of Applied Medical Sciences, Al-Baha University, Al Baha 65779, Saudi Arabia; mohammad.aref@bu.edu.sa (M.A.A.);; 2 Department of Epidemiology and Biostatistics, National Cancer Institute, Cairo University, Cairo 11796, Egypt; 3 Department of Surgery, Faculty of Medicine, Al-Baha University, Al Baha 65779, Saudi Arabia; 4 Public Health Administration, King Fahad Hospital, Ministry of Health, Al Baha 65779, Saudi Arabia; 5 Department of Clinical Pharmacy, Faculty of Pharmacy, Al-Baha University, Al Baha 65779, Saudi Arabia; 6 Faculty of Pharmacy, Al-Baha University, Al Baha 65779, Saudi Arabia; 7 Faculty of Nursing, Al-Baha University, Al Baha 65779, Saudi Arabia; 8 Faculty of Medicine, Al-Baha University, Al Baha 65779, Saudi Arabia

**Keywords:** diabetes, HRQoL, psychological, physical, sexual health, socioeconomic, comorbidity, Al Baha

## Abstract

Background: Type 2 diabetes mellitus (T2DM) presents a significant and escalating public health burden in Saudi Arabia, adversely affecting various dimensions of patients’ well-being. This study aimed to assess the multidimensional quality of life (QoL), also referred to as health-related quality of life (HRQoL) across financial, physical, psychological, and sexual domains among individuals with T2DM in Al Baha, Saudi Arabia, and to compare these outcomes with non-diabetic individuals. Methods: A cross-sectional analytical study was conducted between October 2023 and November 2024 using a structured, self-administered online questionnaire. A total of 948 adults participated, including 495 individuals diagnosed with T2DM and 453 non-diabetic controls. The instrument collected sociodemographic data, disease history, treatment modalities, comorbidities, and QoL indicators across four domains. Results: Diabetic participants demonstrated significantly lower scores in psychological, physical, and sexual QoL domains compared to non-diabetics (*p* < 0.05). Lower psychological QoL was independently associated with younger age and longer disease duration. Physical QoL was significantly influenced by gender, disease duration, and type of treatment, while sexual QoL was negatively impacted by lower educational attainment and presence of comorbidities. A higher financial burden was strongly correlated with reduced psychological and physical QoL (*r* = −0.569 and −0.469, respectively; *p* < 0.001). Conclusions: T2DM is associated with impaired QoL across multiple domains. Targeted, multidimensional interventions are needed to address the physical, psychological, sexual, and financial challenges faced by diabetic individuals in this region.

## 1. Introduction

Diabetes mellitus is a chronic metabolic disorder characterized by hyperglycemia resulting from defects in insulin secretion, insulin action, or both. It is considered one of the most pressing global public health concerns, with an increasing incidence across developed and developing nations. Type 2 diabetes mellitus (T2DM), the most prevalent form, accounts for more than 90% of all diabetes cases worldwide [1]. The condition imposes significant physical and psychological challenges and affects patients’ financial and social well-being, particularly in low- and middle-income countries. In Saudi Arabia, the burden of diabetes is especially alarming. According to the International Diabetes Federation (IDF), the Kingdom ranks among the top ten countries in the world for diabetes prevalence [2]. Lifestyle factors such as sedentary behavior, unhealthy dietary patterns, and increasing obesity rates are major contributors to this public health issue [3]. Importantly, diabetes exerts a multidimensional impact on patients’ lives, affecting their physical health and mental, emotional, and social functioning. Patients often experience depression, anxiety, sexual dysfunction, social isolation, and financial hardship, all of which contribute to a reduced quality of life (QoL) [4].

The World Health Organization (WHO) defines QoL as an individual’s perception of their position in life in the context of their culture, value systems, goals, and expectations [5]. In patients with chronic diseases like diabetes, QoL is increasingly recognized as an essential health outcome, complementing traditional clinical measures such as glycemic control [6]. Health-related quality of life (HRQoL) encompasses various domains, including physical, psychological, sexual, and financial aspects of living with the disease [7]. Multiple factors influence QoL in individuals with diabetes, including disease duration, treatment modality (especially insulin use), the presence of comorbid conditions, gender, educational level, and socioeconomic status [5,8,9]. For example, research suggests that diabetic patients undergoing combined or insulin-based therapy often report lower scores in physical and psychological QoL domains, potentially due to the perceived burden of treatment or disease progression [9]. Moreover, financial costs related to medication, healthcare visits, and loss of productivity can significantly impact patients’ emotional and social well-being [5]. Pharmacological therapies, while essential, may introduce side effects that negatively affect QoL. Non-pharmacological strategies such as diet, exercise, and regular medical follow-up are equally important for long-term management.

Although several studies have examined QoL in diabetic populations, few have comprehensively assessed the interplay of financial, physical, psychological, and sexual domains in the Saudi context, particularly within specific regions such as Al-Baha. Understanding the local determinants of QoL is crucial for developing culturally relevant, patient-centered care strategies that go beyond clinical management. Therefore, the present study aims to evaluate the multidimensional QoL encompassing physical, psychological, sexual, and financial domains among individuals with T2DM residing in Al-Baha, Saudi Arabia, and to compare these outcomes with those of non-diabetic individuals. By identifying key sociodemographic and clinical predictors of impaired QoL, this study aimed to evaluate QoL across physical, psychological, sexual, and financial domains among individuals with T2DM in Al Baha, Saudi Arabia, compared with non-diabetic controls. These efforts are closely aligned with the objectives of Saudi Vision 2030, which prioritizes enhancing the overall QoL through improved healthcare access, chronic disease prevention, and equitable well-being across all regions of the Kingdom.

## 2. Materials and Methods

### 2.1. Study Design and Setting

This study employed a cross-sectional analytical design to evaluate the quality of life (QoL) among individuals with type 2 diabetes mellitus (T2DM) and compare their outcomes with those of non-diabetic participants. Data was collected between 8 October 2023 and 17 November 2024 to capture a broad representation of both newly diagnosed and long-term diabetic cases. The cross-sectional design was chosen for its suitability in examining the relationship between diabetes status and multidimensional QoL at a single time point, enabling the identification of population-level associations that can inform targeted public-health interventions. This approach has been widely and effectively applied in similar QoL investigations among diabetic populations in Saudi Arabia [10,11].

### 2.2. Participants, Sampling, and Sample Size

Participants were recruited using a convenience sampling approach through the online distribution of a structured questionnaire via social media platforms (WhatsApp and Twitter), targeting residents of the Al-Baha region, Saudi Arabia. A total of 948 individuals participated in the study, including 495 adults diagnosed with type 2 diabetes mellitus (T2DM) for at least one year and 453 non-diabetic individuals who served as the control group. Eligible participants were required to be Arabic-speaking adults aged 18 years or older who were able to read and independently complete the survey. Individuals with gestational or type 1 diabetes, as well as those with incomplete responses, were excluded. Both male and female participants were included to ensure gender diversity within the sample. To determine an adequate sample size, calculations were based on findings from a previous study by Gebremedhin et al. [12], which reported that 33.7% of diabetic patients had good QoL. Using this proportion, with a 95% confidence level and a 5% margin of error, the minimum required sample size was estimated to be 344 diabetic participants, indicating that the final sample of 495 diabetic respondents exceeded the minimum requirement and ensured sufficient statistical power.

### 2.3. Instrument Development and Data Collection

The research instrument utilized in this study was specifically developed through a structured and consultative process. A team of public-health experts initiated the development by conducting an extensive literature review to identify relevant domains influencing the quality of life (QoL) in individuals with Type 2 Diabetes Mellitus (T2DM). The questionnaire was designed utilizing established QoL frameworks and previous diabetes-related QoL studies. Draft items were subsequently refined and reviewed by specialists to guarantee clarity, cultural appropriateness, and content validity, and the instrument was pilot tested to confirm reliability before data collection began.

The questionnaire was administered online using Google Forms, with the survey link disseminated widely through social media platforms (WhatsApp and Twitter) and email to reach adult residents of Al-Baha, Saudi Arabia. Before commencing the survey, participants were informed of the study’s objectives and provided digital informed consent. The final instrument was designed to assess multidimensional QoL and its determinants, capturing sociodemographic, clinical, and QoL data. The core QoL assessment consisted of 15 items divided across three domains psychological (8 items), physical (4 items), and sexual (3 items) with each rated on a four-point Likert scale (1 = “never” to 4 = “always”), where higher scores reflected better perceived QoL. Additionally, the instrument included five dedicated questions for the evaluation of the financial burden of diabetes, distinguishing this dimension by the fact that higher scores indicated greater perceived economic strain on daily life.

### 2.4. Pilot Testing and Reliability

Before rolling out the questionnaire to the full sample, a pilot test was conducted with 15 participants to evaluate the clarity and acceptability of the questionnaire. Based on the feedback received, minor modifications were made. To ensure scientific rigor, five faculty members established content validity through expert review. The internal consistency of the QOL domains was assessed using Cronbach’s alpha, with reliability coefficients as follows: 0.78 for the psychological domain, 0.82 for the physical domain, and 0.86 for the sexual domain, indicating satisfactory to high reliability across all subscales.

### 2.5. Ethical Considerations

The study was approved by the Research Ethics Committee of Al Baha University (Ref: 1445/45127769; Approval Date: 28 August 2023) and conducted in accordance with the Declaration of Helsinki. Digital informed consent was obtained from all participants prior to data collection. All data were handled in compliance with international Data Protection principles and the General Data Protection Regulation (GDPR, EU 2016/679) to ensure confidentiality and prevent unauthorized access.

### 2.6. Data Analysis

Data was analyzed using IBM SPSS Statistics version 26.0 (IBM Corp., Armonk, NY, USA). Descriptive statistics summarize sample characteristics. Normality was assessed using the Kolmogorov–Smirnov test. Inferential analyses included Chi-square tests for categorical variables, Mann–Whitney U and Kruskal–Wallis tests for non-parametric group comparisons, with Bonferroni-adjusted post hoc analyses. Spearman’s correlation assessed associations between continuous variables. Variables significant in univariate analysis were entered into a multiple linear regression model to identify independent QoL predictors. Statistical significance was set at *p* ≤ 0.05.

## 3. Results

### 3.1. Sociodemographic Profile of Study Participants

The study sample comprised 948 participants, including 495 individuals with diagnosed T2DM (52.2%) and 453 non-diabetic individuals (47.8%). The mean age of diabetic participants was 40.7 ± 17.2 years, significantly higher than the average age of non-diabetic participants (27.1 ± 10.6 years). Females represented the majority in both groups, comprising 55.8% of the diabetic group and 68.0% of the non-diabetic group. Regarding marital status, 57% of diabetic participants were married, compared to 31.1% in the non-diabetic group. Educational attainment varied between groups, with 50.3% of diabetic individuals and 75.7% of non-diabetic individuals reporting a university-level education. Full sociodemographic distributions are detailed in Table 1.

### 3.2. Comorbidities, Diabetes Duration, and Treatment Modalities

Among diabetic participants, the most frequently reported comorbid conditions were hypercholesterolemia (54.7%), hypertension (47.1%), neuropathy (40.8%), and vision impairment (40.4%). Compared to non-diabetic individuals, diabetic participants exhibited significantly higher rates of hypercholesterolemia, hypertension, nerve problems, vision problems, erectile dysfunction, hypoactive sexual desire disorder, and cervical infections (all *p* < 0.05) (Table 2). Regarding disease duration, 51.5% of diabetic participants were diagnosed for six years or less, while 48.5% had lived with the disease for more than six years. Regarding management, 37.8% of participants were treated with oral hypoglycemic agents, 24.2% with insulin injections, 22% with a combination of oral agents and insulin, and 16% managed their diabetes through dietary modification alone. Additionally, 72.1% reported taking vitamin supplements. Symptoms of hypoglycemia were reported at least once per month by 32.1% of diabetic participants (Table 3).

### 3.3. Determinants of Quality of Life in Diabetic Participants

**Figure 1:**
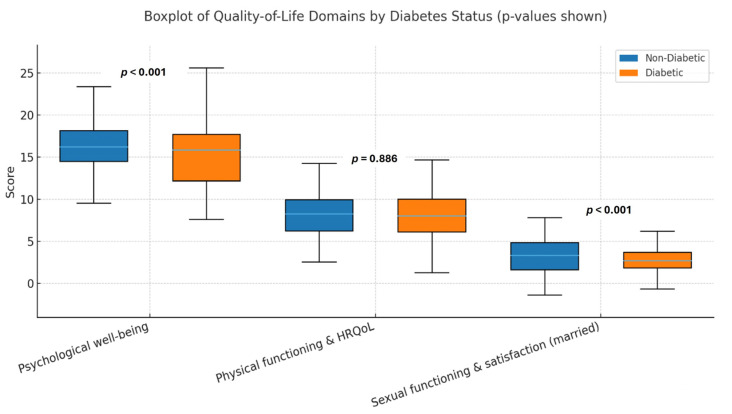
Comparison of quality-of-life domains between diabetic and non-diabetic participants. *p*-values were calculated using the Mann–Whitney U test.

Diabetic participants exhibited significantly lower scores in both psychological and physical QoL domains compared to non-diabetic individuals (*p* < 0.05) (Figure 1).

Psychological QoL was significantly influenced by several demographic and clinical factors. Participants aged ≥35 years reported higher mean psychological QoL scores compared to those aged <35 years (16.3 ± 3.9 vs. 15.2 ± 4.4; *p* = 0.034). Male participants demonstrated significantly better psychological QoL than females (*p* = 0.011). Additionally, a disease duration exceeding six years was associated with lower psychological QoL (*p* = 0.047), as was the use of vitamin supplementation (*p* = 0.009). Physical QoL was significantly higher among male participants and Saudi nationals (*p* < 0.05). Educational attainment showed a strong association with physical QoL (*p* < 0.001), where post hoc analysis revealed significantly lower physical QoL scores among illiterate individuals compared to those with secondary, high school, or university education (adjusted *p* < 0.001, 0.005, and 0.001, respectively). Marital status was also significantly associated with physical QoL (*p* < 0.001); widowed participants had lower scores than single, married, and divorced counterparts (adjusted *p* = 0.002, <0.001, and <0.0001, respectively). Regarding treatment modality, participants treated with oral agents, insulin, or combined therapy had significantly lower physical QoL scores compared to those managing their condition through dietary modification (adjusted *p* < 0.001, 0.001, and <0.0001, respectively). Moreover, individuals with a disease duration greater than six years had significantly reduced physical QoL (*p* < 0.001). The presence of multiple comorbidities was also negatively associated with physical QoL, with significantly lower scores observed among those with more than one comorbid condition compared to those without (adjusted *p* < 0.001). Sexual QoL was significantly associated with both education level (*p* = 0.008) and comorbidity status (*p* < 0.001). Participants with postgraduate education had significantly higher sexual QoL compared to those with a high school education (adjusted *p* = 0.039). Individuals without comorbidities had significantly better sexual QoL scores than those with one or more comorbid conditions (adjusted *p* = 0.001 and <0.001, respectively) (Table 4).

Multivariate linear regression analysis identified the independent predictors of each QoL domain. Age and duration of diabetes independently predicted psychological QoL. Physical QoL was independently associated with disease duration, treatment modality, and gender. Educational level and comorbidity status emerged as independent predictors of sexual QoL (Table 5).

### 3.4. Financial Burden and Its Association with Quality of Life

The perceived financial burden among diabetic participants was notably high, with a mean score of 8.5 ± 3.8, indicating a moderate to substantial economic impact related to disease management (Table 6). Correlational analyses revealed a statistically significant inverse relationship between the physical QOL, psychological QOL domains, and financial burden. Specifically, higher financial burden scores were associated with lower psychological QoL (Spearman’s *r* = −0.569, *p* < 0.001) and lower physical QoL (Spearman’s *r* = −0.469, *p* < 0.001), suggesting that economic strain may substantially diminish mental well-being and physical functioning in individuals with T2DM. In contrast, no statistically significant correlation was observed between financial burden and sexual QoL (*p* > 0.05), indicating that perceived economic hardship may have limited direct influence on patients’ sexual health perceptions within this sample. These findings underscore the multidimensional impact of financial stress on diabetic patients’ well-being. The results highlight the importance of integrating financial counseling and access to cost-effective care into diabetes management strategies, especially in regions where out-of-pocket health expenditures remain a barrier to sustained treatment adherence and QoL.

## 4. Discussion

This study provides a comprehensive assessment of the QoL among individuals living with T2DM, revealing a significantly lower QoL across multiple domains, including psychological, physical, sexual, and financial well-being, compared to non-diabetic individuals. These findings emphasize the complex and multidimensional burden that T2DM places on patients’ daily lives, extending beyond metabolic control to affect emotional resilience, physical functioning, and socioeconomic stability.

Although age and gender differed between diabetic and non-diabetic participants, subgroup analyses indicated that the lower QoL scores among individuals with diabetes persisted within comparable demographic strata, suggesting that these differences were not solely attributable to sociodemographic variation.

Psychological QoL was particularly reduced among diabetic participants, especially in younger individuals, females, and those with longer disease duration. Physical QoL was also significantly compromised, notably among individuals receiving pharmacological therapy, those with lower educational attainment, and patients managing additional comorbid conditions. Similarly, sexual QoL was found to be lower in participants with limited education and multiple health issues. In contrast, non-diabetic participants reported more stable and favorable QoL outcomes across all domains. These findings are consistent with previous research showing that individuals with diabetes tend to experience diminished QoL relative to those without chronic illness, although the underlying causes are often multifactorial [10,13]. Routine incorporation of QoL assessment into diabetes care could guide individualized treatment planning. Psychosocial support, financial counseling, and structured diabetes education may reduce disease burden. Digital health innovations and culturally adapted lifestyle programs may further enhance patient outcomes. The marked impact on physical QoL observed in our study parallels findings from Ghana and Mexico, where physical limitations emerged as a primary concern among diabetic patients [13]. These impairments may be attributed to the cumulative effects of long-term complications, medication side effects, and the progressive nature of the disease Gender differences in QoL were also evident, with female participants reporting significantly lower scores in both psychological and physical domains. These results corroborate earlier studies, including regional data from the Eastern Province of Saudi Arabia, which revealed poorer QoL among women with diabetes [10,11]. Sociocultural factors, such as gender roles and health-seeking behavior in Arab societies, may partially explain these disparities, with female sex recognized as an independent predictor of reduced QoL [11].

The 35-year age cutoff was selected based on the distribution of our data and its ability to differentiate QoL outcomes across age groups. Alternative cutoffs at 45 or 65 years did not produce significant variation within our sample, suggesting that the 35-year threshold was the most appropriate for detecting relevant age-related differences.

Interestingly, older participants (≥35 years) in our sample reported better psychological QoL than younger individuals, a finding that contrasts with previous studies where younger age was associated with improved well-being [11]. This discrepancy may reflect differences in population characteristics or psychosocial adaptation, where older adults develop better coping mechanisms over time.

Disease duration was a critical determinant of QoL. Participants living with diabetes for more than six years reported significantly lower psychological and physical QoL, reinforcing findings from Muze et al. [14] and others [13,15]. However, Mishra et al. [16] reported better outcomes among patients with longer disease durations, suggesting that sustained social support and lifestyle adjustments might mitigate the negative effects in some individuals.

Treatment modality also influenced QoL outcomes. Those on insulin, oral hypoglycemic agents, or combination therapy had significantly lower physical QoL compared to patients managing diabetes through diet alone. This aligns with findings by Homady et al. [17], who noted that pharmacological treatment, while clinically necessary, may contribute to reduced QoL due to regimen complexity, perceived illness severity, and adverse effects.

Comorbidities were strongly associated with lower QoL, echoing findings by Didarloo et al. [18] and another Saudi-based study [10]. Individuals with multiple comorbid conditions likely face greater health challenges, increased medication burdens, and more frequent healthcare visits, all of which contribute to diminished overall well-being. Sexual QoL was also adversely affected in diabetic patients, consistent with findings from Keshtkar et al. [19], who emphasized the negative influence of diabetes on sexual functioning and satisfaction. Importantly, the financial burden emerged as a significant factor linked to QoL. Higher perceived financial strain was associated with lower scores in both physical and psychological domains. This finding is supported by Kim et al. [20] and others [21,22], who identified financial hardship as a risk factor for reduced QoL in diabetic populations. The cumulative cost of medications, medical consultations, and indirect losses such as work absenteeism likely to contribute to this burden. Collectively, these findings illustrate that diabetes is not solely a metabolic disorder but a life-altering condition that impacts individuals on emotional, physical, social, and economic levels. Addressing these interrelated domains is essential for delivering holistic and effective care.

The multidimensional burden observed across psychological, physical, sexual, and financial domains highlights the urgent need for integrated, patient-centered management frameworks. Future research should adopt longitudinal and interventional designs to evaluate how psychosocial support systems, digital and telehealth-based care models, and targeted financial counseling can alleviate these challenges. Such approaches would strengthen causal evidence, enhance real-world applicability, and guide region-specific health policy and resource allocation in alignment with Saudi Vision 2030 objectives.

Findings from this study can assist public health practitioners in tailoring intervention programs, particularly within primary healthcare settings, to address psychosocial, physical, and financial burdens among diabetic individuals. These insights can guide workforce training and resource allocation strategies across underserved regions.

The implications of this study are particularly relevant within the framework of Saudi Vision 2030, which emphasizes improving population health and enhancing QoL nationwide. By identifying specific determinants of QoL among individuals with T2DM, this research contributes to evidence-based strategies aimed at advancing health equity, preventive care, and patient-centered outcomes. Furthermore, the study addresses a notable knowledge gap in the Al-Baha region, an underrepresented area with unique healthcare challenges and sociodemographic characteristics. The insights generated here can inform the design of targeted interventions and resource allocation strategies tailored to diabetic populations in underserved regions. The relatively younger mean age of participants also reflects the demographic profile of the Al-Baha region, where younger adults constitute a substantial proportion of individuals affected by type 2 diabetes [23].

Methodologically, this study is strengthened by its large sample size, the inclusion of a non-diabetic control group, and its multidimensional evaluation of QoL domains. These design features enhance the robustness and comparative depth of the analysis. Future strategies should also address lifestyle interventions and explore innovative approaches such as mobile health applications and telemedicine to strengthen patient-centered care. Nonetheless, certain limitations should be acknowledged. The cross-sectional nature of the study precludes causal inference, and the reliance on self-reported data may introduce recall or social desirability bias. Additionally, findings may not be generalizable to other regions of Saudi Arabia due to potential variations in cultural practices and healthcare infrastructure. Considering these findings, it is recommended that psychosocial and financial support services be integrated into standard diabetes care. High-risk subgroups, including younger adults, females, and those receiving pharmacological treatment should be prioritized for targeted interventions. Incorporating routine QoL assessments into clinical practice could aid in the early detection of at-risk individuals and guide personalized management strategies. These approaches not only align with national health objectives but also have the potential to significantly enhance the lived experiences and long-term outcomes of individuals with diabetes.

## 5. Conclusions

This study showed that individuals with type 2 diabetes mellitus in Al Baha experienced lower quality of life across psychological, physical, sexual, and financial domains compared with non-diabetic participants. These results highlight the wide-ranging impact of diabetes beyond blood sugar control. Future research should focus on innovative strategies, including digital health technologies, structured lifestyle programs, and culturally adapted support systems, to improve patient-centered outcomes and promote long-term well-being.

## Figures and Tables

**Table 1:** Sociodemographic characteristics of the study participants by diabetes status.

		Diabetes		
	All (*n* = 948)	No (*n* = 453)	Yes (*n* = 495)	*p*-Value
**Age (years)**	34.2 ± 15.9	27.1 ± 10.6	40.7 ± 17.2	<0.001
**Age**				
<35 years	558 (58.9%)	354 (78.1%)	204 (41.2%)	<0.001
≥35 years	390 (41.1%)	99 (21.9%)	291 (58.8%)	
**Gender**				
Female	584 (61.6%)	308 (68%)	276 (55.8%)	<0.001
Male	364 (38.4%)	145 (32%)	219 (44.2%)	
**Nationality**				
Non-Saudi	20 (2.1%)	11 (2.4%)	9 (1.8%)	0.514
Saudi	928 (97.9%)	442 (97.6%)	486 (98.2%)	
**Marital status**				
Single	470 (49.6%)	302 (66.7%)	168 (33.9%)	<0.001
Married	423 (44.6%)	141 (31.1%)	282 (57%)	
Divorced	24 (2.5%)	6 (1.3%)	18 (3.6%)	
Widow	31 (3.3%)	4 (0.9%)	27 (5.5%)	
**Education**				
Illiterate	33 (3.5%)	0 (0%)	33 (6.7%)	<0.001
Primary	30 (3.2%)	3 (0.7%)	27 (5.5%)	
Secondary	48 (5.1%)	3 (0.7%)	45 (9.1%)	
High School	204 (21.5%)	90 (19.9%)	114 (23%)	
Undergraduate	592 (62.4%)	343 (75.7%)	249 (50.3%)	
Postgraduate	41 (4.3%)	14 (3.1%)	27 (5.5%)	
**Employment**				
No	660 (69.6%)	345 (76.2%)	315 (63.6%)	<0.001
Yes	288 (30.4%)	108 (23.8%)	180 (36.4%)	

Frequencies and percentages are reported for categorical variables and means ± standard deviations are reported for continuous variables. Group comparisons were conducted to assess differences between diabetic and non-diabetic individuals. *p*-values were calculated using the Chi-square test.

**Table 2:** Distribution of self-reported comorbid conditions among study participants according to diabetes status.

		Diabetes		
	All (*n* = 948)	No (*n* = 453)	Yes (*n* = 495)	*p*-Value
Hypercholesterolemia	186 (19.6%)	18 (4%)	168 (33.9%)	<0.001
Hypertension	160 (16.9%)	19 (4.2%)	141 (28.5%)	<0.001
Peripheral neuropathy	142 (15%)	28 (6.2%)	114 (23%)	<0.001
Visual impairment	91 (9.6%)	13 (2.9%)	78 (15.8%)	<0.001
Erectile dysfunction	60 (6.3%)	6 (1.3%)	54 (10.9%)	<0.001
Endocrine disorders	63 (6.6%)	15 (3.3%)	48 (9.7%)	<0.001
Cardiovascular disease	54 (5.7%)	9 (2%)	45 (9.1%)	<0.001
Cervical infection	33 (3.5%)	9 (2.0%)	24 (4.8%)	0.016
Chronic kidney disease (CKD)	6 (0.6%)	3 (0.7%)	3 (0.6%)	0.913
Hypoactive sexual desire disorder (HSDD)	39 (4.1%)	6 (1.3%)	33 (6.7%)	<0.001
Peripheral vascular disease (PVD)	6 (0.6%)	0 (0%)	6 (1.2%)	0.019
Other medical conditions	20 (2.1%)	5 (1.1%)	15 (3%)	0.039

Statistical comparisons between diabetic and non-diabetic groups were conducted using the Chi-square test, with a *p*-value < 0.05 considered statistically significant.

**Table 3:** Clinical characteristics, duration of diabetes, and treatment patterns among individuals with type 2 diabetes mellitus (*n* = 495).

	Frequency (%)
**Years of having diabetes**	
Short-term duration (≤6 years)	255 (51.5%)
Long-term duration (>6 years)	240 (48.5%)
**Type of Diabetes Management**	
Dietary management only	79 (16%)
Oral hypoglycemic agents only	187 (37.8%)
Insulin monotherapy	120 (24.2%)
Combination therapy (oral agents + insulin)	109 (22%)
**Type of Oral Hypoglycemic Agents Used (*n* = 296)**	
Single oral agent	146 (49.3%)
Multiple oral agents	150 (50.7%)
**Use of vitamin supplements**	
Yes	357 (72.1%)
No	138 (27.9%)
**Use of complementary or traditional therapies**	
Yes	63 (12.7%)
No	432 (87.3%)
**Times for experiencing hypoglycemia symptoms**	
No hypoglycemic symptoms	150 (30.3%)
Daily episodes	27 (5.5%)
Weekly episodes	96 (19.4%)
2–3 episodes per week	63 (12.7%)
Monthly episodes	159 (32.1%)

**Table 4:** Sociodemographic and clinical factors associated with quality-of-life domains among individuals with type 2 diabetes mellitus.

	Quality of Life					
	Psychological	*p*-Value	Physical	*p*-Value	Sexual	*p*-Value
**Age groups**						
<35 years	15.2 ± 4.4	0.034	7.9 ± 3.2	0.627	2.7 ± 1.9	0.515
≥35 years	16.3 ± 3.9		7.9 ± 3.03		2.9 ± 1.5	
**Gender**						
Female	15.4 ± 4.15	0.011	7.6 ± 3.2	0.006	3.03 ± 1.5	0.023
Male	16.5 ± 4.10		8.5 ± 2.8		2.7 ± 1.6	
**Nationality**						
Non-Saudi	11 ± 6.5	0.079	5.5 ± 0.6	0.015	3 ± 0.2	0.738
Saudi	15.9 ± 4.1		7.9 ± 3.1		2.9 ± 1.6	
**Marital status**						
Single	15.3 ± 4.5	0.126	7.7 ± 3.2	<0.001		
Married	16.3 ± 3.5		8.3 ± 2.7			
Divorced	14.8 ± 6.3		9 ± 4.1			
Widow	15 ± 5.3		4.9 ± 2.9			
**Education**						
Illiterate	13.9 ± 5.81	0.061	5.3 ± 3.5	<0.001	2.6 ± 0.5	0.008
Primary	16.7 ± 4.19		7 ± 2.4		2.3 ± 1.4	
Secondary	16.8 ± 3.44		9.2 ± 2.9		2.5 ± 1.1	
High School	16.9 ± 3.4		7.9 ± 3.5		2.7 ± 1.8	
Undergraduate	15.6 ± 3.9		8.2 ± 2.7		3 ± 1.8	
Postgraduate	14.4 ± 6.22		6.8 ± 2.8		4 ± 1.31	
**Employment**						
No	16.1 ± 4.1	0.157	8.02 ± 3.2	0.366	2.8 ± 1.4	0.517
Yes	15.2 ± 4.2		7.7 ± 2.9		2.9 ± 1.8	
**Years of having diabetes**						
Short-term duration (≤6 years)	16.1 ± 3.8	0.047	8.7 ± 2.9	<0.001	2.9 ± 1.7	0.789
Long-term duration (>6 years)	15.4 ± 4.5		6.9 ± 3.2		2.8 ± 1.4	
**Type of treatment**						
Dietary management only	16.5 ± 4.42	0.152	9.5 ± 2.1	<0.001	2.3 ± 2.03	0.119
Oral hypoglycemic agents only	15.9 ± 3.80		8.03 ± 3.3		3.03 ± 1.31	
Insulin monotherapy	15.7 ± 4.20		7.4 ± 3.1		3.2 ± 1.69	
Combination therapy (oral agents + insulin)	15.1 ± 4.4		7.1 ± 3.2		2.7 ± 1.7	
**Type of Oral Hypoglycemic Agents Used (*n* = 296)**						
Single oral agent	15.7 ± 4.5	0.589	7.7 ± 3.4	0.653	2.9 ± 1.6	0.676
Multiple oral agents	15.5 ± 3.5		7.6 ± 3.2		2.8 ± 1.3	
**Use of vitamin supplements**						
No	16.7 ± 3.9	0.009	8.3 ± 2.8	0.097	2.9 ± 1.2	0.618
Yes	15.4 ± 4.2		7.8 ± 3.3		2.8 ± 1.8	
**Comorbidity**						
Absence of comorbid conditions	16.1 ± 3.8	0.774	8.6 ± 2.7	<0.001	3.5 ± 1.3	<0.001
Single comorbid condition	15.9 ± 3.8		7.9 ± 2.9		2.7 ± 1.7	
Multiple comorbid conditions	15.3 ± 4.5		6.9 ± 3.6		2.5 ± 1.5	

Group comparisons were conducted using non-parametric tests, with Bonferroni-adjusted *p*-values reported for post hoc analyses. Statistically significant values (*p* < 0.05) indicate variables that may predict impaired quality of life in specific domains.

**Table 5:** Multivariate linear regression analysis of factors associated with quality-of-life domains among individuals with type 2 diabetes mellitus.

**Psychological Well-Being**			
	**β**	**95% CI for β**	***p*-Value**
Age groups	0.138	0.5, 1.9	0.007
Years of having diabetes	−0.100	−1.7, −0.004	0.049
**Physical functioning and health-related quality of life**			
	**β**	**95% CI for β**	***p*-value**
Years of having diabetes	−0.226	−2.042, −0.789	<0.001
Type of Diabetes Management	−0.176	−0.841, −0.229	0.001
Gender	0.160	0.423, 1.661	0.001
**Sexual functioning and satisfaction among married individuals**			
	**β**	**95% CI for β**	***p*-value**
Education	0.156	0.017, 0.352	0.031
Comorbidity	−0.144	−0.621, −0.006	0.046

The table reports standardized regression coefficients (β), 95% confidence intervals (CI), and associated *p*-values. Variables with *p* < 0.05 were considered statistically significant predictors of QoL in the respective domains.

**Table 6:** Correlation between financial burden and quality of life domains among individuals with type 2 diabetes mellitus.

	Financial Burden Score	
	8.5 ± 3.8	
	***r***	***p*-value**
Psychological well-being	−0.569	<0.001
Physical functioning and health-related quality of life	−0.469	<0.001
Sexual functioning and satisfaction among married individuals	0.036	0.509

Correlation was assessed using Spearman’s rank correlation coefficient (r).

## Data Availability

The datasets analyzed during the current study are available from the corresponding author on reasonable request.

## Abbreviations

The following abbreviations are used in this manuscript:

T2DM	Type 2 Diabetes Mellitus
QoL	Quality of Life
HRQoL	Health-Related Quality of Life
WHO	World Health Organization
IDF	International Diabetes Federation
SPSS	Statistical Package for the Social Sciences
SD	Standard Deviation
BMI	Body Mass Index
DM	Diabetes Mellitus
CI	Confidence Interval
PVD	Peripheral Vascular Disease
